# Radiofrequency ablation combined with transarterial chemoembolization in treatment of hepatocellular carcinoma adjacent to the second hepatic hilus

**DOI:** 10.1007/s00261-021-03304-4

**Published:** 2021-10-11

**Authors:** Meng-Li Chen, Hai-Liang Li, Chen-Yang Guo, Hao Zhang, Hang Yuan, Zhen Li, Jung-Hoon Park, Hong-Tao Hu

**Affiliations:** 1 Minimally Invasive and Interventional Department, Affiliated Cancer Hospital of Zhengzhou University, Henan Cancer Hospital, 127 Dongming Road, Zhengzhou, 450008 Henan China; 2 Department of Interventional Radiology, First Affiliated Hospital of Zhengzhou University, Zhengzhou, 450052 Henan China; 3 Biomedical Engineering Research Center, Asan Institute for Life Sciences, Asan Medical Center, 88 Olympic-ro 43-gil, Songpa-gu, Seoul, 05505 Republic of Korea

**Keywords:** Radiofrequency ablation (RFA), Transarterial chemoembolization (TACE), Hepatocellular carcinoma, Second hepatic hilus

## Abstract

**Purpose:**

To explore the efficacy and safety of using radiofrequency ablation (RFA) combined with transarterial chemoembolization (TACE) for treating hepatocellular carcinoma (HCC) adjacent to the second hepatic hilus.

**Methods:**

Between February 2011 and June 2013, 17 patients with HCC underwent combination therapy of TACE and RFA under DSA and CT guidance at our institution. The 17 patients had a total of 23 hepatic tumors, 17 of which were adjacent to the second hepatic hilus.

**Results:**

TACE combined with RFA was performed successfully in all 17 patients with no mortalities or major morbidities. During the 1-month follow-up, tumors of 15 patients (88.2%) were completely ablated after one therapy session and 2 patients had detectable tumor residue. During the follow-up time period (range 6–52 months), local tumor progression developed in 1 patient (1/17, 5.9%) and both local tumor progression and new tumors appeared in 1 patient (1/17, 5.9%). Also, new tumors developed in the untreated portions of the liver in 8 patients (8/17, 47.1%). No distant metastasis was found. Of the 17 patients, 6 (35.3%) died due to tumor progression (3/17, 17.6%), liver failure (2/17, 11.8%), or massive hemorrhage of the gastrointestinal tract (1/17, 5.9%). The overall survival rates were 94.1% (16/17), 82.4% (14/17), and 61.8% (11/17) at 12, 18, and 24 months, respectively, and the median survival time was 25 months (95% CI 18–27).

**Conclusion:**

Treatment using combination of TACE and RFA is an effective and safe therapeutic strategy for treating HCC with tumor(s) adjacent to the second hepatic hilus.

## Introduction

Traditionally, hepatic resection and transplantation have been considered the treatments of choice for treatment of patients with hepatocellular carcinoma (HCC) [1]. However, radiofrequency ablation (RFA) is becoming a powerful local treatment for patients with small size HCC (diameter less than 3 cm) [2, 3]. Recently, RFA has become one popular and effective method of percutaneous ablation therapy, especially for the patients with early HCC cases of either single (1 nodule, ≤ 50 mm) or multiple lesions (up to 3 nodules, ≤ 30 mm each). Compared with hepatic resection, RFA demonstrated higher efficacy and safety due to it being less invasive, preserving liver function, being simpler to perform, and requiring a shorter hospitalization [4–7]. The combined application of transarterial chemoembolization (TACE) with RFA is appealing [8]. TACE can decrease blood flow to the tumor(s) and reinforce subsequent RFA efficacy due to less heat being lost through convection [9]. The reduction of heat sink leads to the enlargement of the ablation zones, which can improve prognosis, especially for large tumors. Several studies have mentioned possible synergistic cytotoxic effects of TACE with RFA for HCC [10, 11].

When the tumor is adjacent to critical structures, such as near the hilar bile duct and blood vessels adjacent to the first or second hepatic hilus or the caudate lobe, it cannot be resected by surgery as is the typical treatment; it can be very difficult for surgical procedures to effectively remove the tumor due to the difficult anatomical location [12]. Previous reports have verified that HCC located in the caudate lobe tends to be difficult to treat because the tumor is enclosed by the hepatic vein, inferior vena cava, and bile ducts [13, 14]. Similarly, RFA therapy for tumors adjacent to the second hepatic hilum is also difficult, due to the heat sink effect that causes damage to the surrounding bile ducts and vein. Therefore, a tumor adjacent to the second hepatic hilus treated by RFA has still not been reported except for a single case reported by Kai et al*.* [15]. It was previously reported that ultrasound-guided ablation was used to treat HCC in difficult locations, but the incidence of complications of tumors in difficult locations was higher than those in accessible locations. The reason is that the location of the lesion is deep, and it is difficult to clearly show the lesion and its surrounding structure. However, CT guidance can avoid these shortcomings and achieve intuitive, clear, and accurate positioning [16]. In our research, we retrospectively evaluated the efficacy and safety of using RFA combined with TACE to treat HCC that is adjacent to the second hepatic hilus in 17 patients.

## Methods

Between February 2014 and June 2016, 287 patients at our hospital underwent a combination strategy of therapeutic TACE and RFA to treat HCC. The diagnosis of HCC was made according to the diagnostic criteria used by the European Association for the Study of the Liver [17]. These diagnostic criteria are defined as either two imaging techniques showing typical features of HCC, positive findings on one imaging study together with an α-fetoprotein level of more than 400 ng/mL, or a histologic diagnosis of HCC. All patients signed an informed consent and gave permission to use their clinical data for our research. Our research protocol conformed to the ethical guidelines of the World Medical Association Declaration of Helsinki and was approved by our Institutional Review Board.

**Fig. 1:**
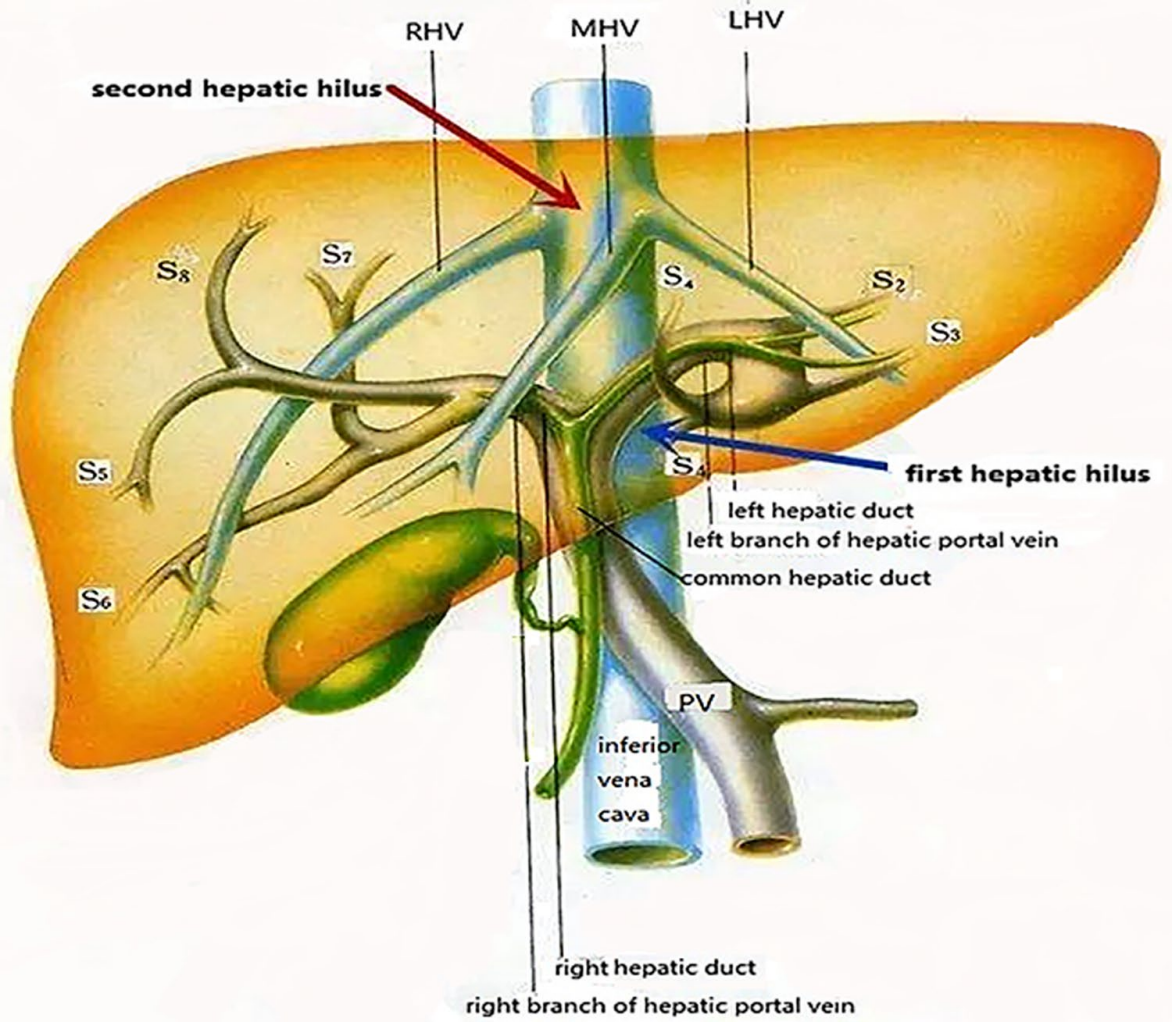
Illustration of the first and second hepatic hilus

According the reference, segments II, IVa, VII, and VIII of the hepatic tumors within 5.0 cm from the root of the main hepatic vein were defined as tumors adjacent to the second hepatic hilus [18]. In this research, we defined the “tumors adjacent to the second hepatic hilus” as the hepatic tumors within 1.0 cm from the main hepatic vein. All patients having a single lesion (1 nodule, ≤ 50 mm) or multiple lesions (up to 3 nodules, ≤ 30 mm each) of the liver and at least one lesion adjacent to the second hepatic hilus (Fig. 1) were included in this study. Patients with extrahepatic metastasis, Child–Pugh class C liver function, and/or uncontrollable ascites were excluded. Based on the previous research of advanced HCC with portal vein tumor thrombosis (PVTT) [19, 20], patients with portal vein thrombosis were also excluded.

Of the 287 patients, 17 patients complicit with the inclusion criteria were included in this study (16 males and 1 female; mean age of 57 years; age range of 33–78 years).

Before treatment, all of the patients underwent a routine physical examination, laboratory testing, and radiologic imaging studies. Imaging studies including a chest CT and a contrast-enhanced abdominal CT scan, or epigastric magnetic resonance imaging were performed within the 2 weeks before TACE.

### TACE procedure

The combined TACE and RFA procedures were performed on an inpatient basis in all tumors. Two interventional radiologists (each one who have over 15 years of experience in interventional radiology procedures) performed the combined TACE and RFA procedures.

TACE was performed with a 5 French RH catheter (Cook, Bloomington, Ind), a Cobra catheter (Cook), or a microcatheter (Progreat, Terumo, Tokyo, Japan) being inserted as carefully as possible through the lobar, segmental, or subsegmental arteries, depending on both the tumor location and the hepatic functional reserve of the patient. To start, an emulsion of 2–12 ml of lipiodol (Lipiodol, Guerbet, Aulnay-Sous-Bois, France), 60–90 mg of cisplatin, and 20–40 mg of doxorubicin hydrochloride was injected into the feeding artery. The dosage of lipiodol, cisplatin, and doxorubicin was based on tumor size and vascularity, the presence or absence of an arterioportal shunt, and underlying liver function. Once the emulsion injection was complete, gelatin sponge particles (300–500 µm) mixed with contrast medium were administered into the feeding arteries until stoppage of arterial flow was achieved.

### RFA procedure

Subsequent RFA was performed as soon as possible following TACE. All 17 patients underwent RFA within the 7 days (3–7 days) proceeding TACE.

**Fig. 2:**
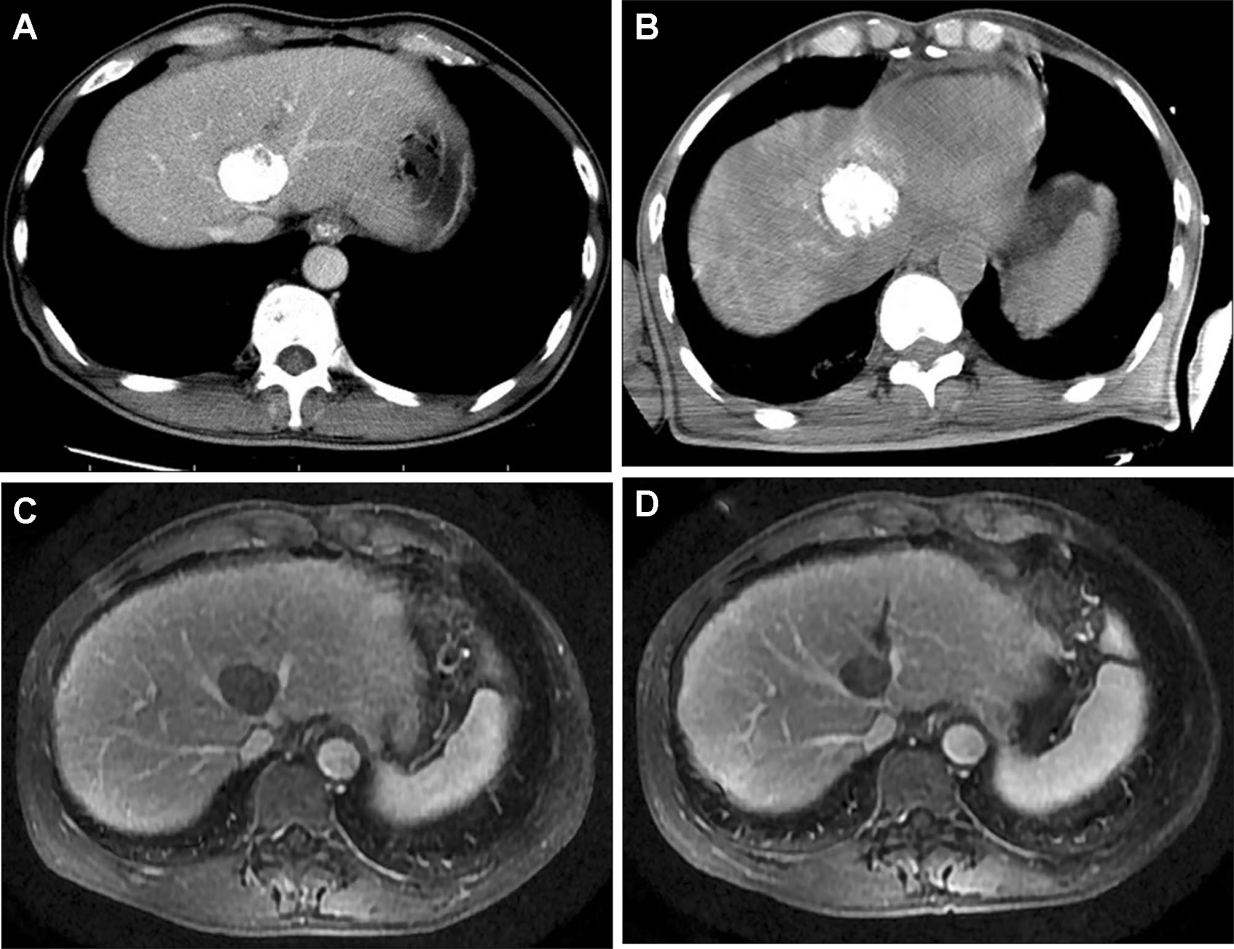
A 60-year-old man with a 47 mm HCC tumor who underwent combination TACE and RFA treatment. **A** Five days after the TACE procedure, a CT scan was obtained before the RFA procedure. The scan showed a good iodine deposition in the tumor. **B** During the 1-month follow-up, the contrast-enhanced CT scan showed a decrease in tumor size. **C** An enhanced MR image 3 months post treatment showed that there was no tumor survival or vascular injury around the tumor. **D** 27 months post procedure, the MR image showed a maintained decrease in tumor size and no tumor recurrence

RFA was performed percutaneously under CT guidance. A commercially available RFA system (RITA 1500X RF generator, RITA Medical Systems, California) and standard technique were used in this study. All patients were in a supine position while under general anesthesia, and grounding was achieved by attaching two pads to the patient’s thighs. The patients’ heart rate, blood pressure, and oxygen saturation were monitored during the procedure. A preprocedural CT scan of the target area was obtained and all lesions were localized with CT images (Fig. 2A). After confirming the path of the needle with imaging, the RF needles were inserted into the tumor with CT guidance and the electrode was advanced through the catheter. The appropriate RF energy level was achieved by activating the generator, and the average temperature was maintained at 100 °C. First, the electrodes were advanced by 2 cm, and then the electrode needles were advanced and gradually unfolded to 3, 4, or 5 cm until the electrodes crossed the tumor boundary in the target range for ablation. For each intermediate step, RF energy was delivered for 5 min, and in the final step, 7 to 10 min of RF energy was provided. The ablation target range was intended to cover the entirety of the tumor as well as ≥ 5 mm of the surrounding tissue, depending on the adjacent vessels or bile duct. During ablation, the temperature was monitored with a thermocouple in the electrode. To prevent bleeding and tumor seeding, when withdrawing the RFA electrode, we routinely performed tissue ablation of the electrode path in all patients when completing the first ablation. If necessary, the electrode was then moved to a second predetermined location and the electrode tines were reinserted to overlap the ablation procedure. The patients did not receive a preventive antibiotic before or after the RFA procedure.

### Follow-up

The follow-up was performed on an outpatient basis by three interventional radiologists. The patients were followed up about 1 month after treatment to evaluate the effect by enhanced CT or MRI and laboratory tests measuring tumor markers such as α-fetoprotein. After two consecutive months without progression or recurrence, the follow-up protocols changed into a routine physical examination, laboratory tests measuring tumor markers such as α-fetoprotein every month, and three-phase contrast-enhanced CT or MRI studies every 3–4 months (Fig. 2B–D). We use the mRECIST criteria to evaluate efficacy [21]. Complete response (CR) was determined as complete ablation. Complete ablation was defined as that enhanced CT or MRI scan showed no enhancement of tumor ablation lesions in arterial phase, suggesting complete necrosis of tumor. Primary therapeutic success was defined as achieving a complete ablation of the target tumor. Local tumor progression was defined as the appearance of tumor foci at the edge of the ablation zone after at least one contrast-enhanced follow-up study used imaging standards to record sufficient ablation of the target tumor and the surrounding ablation margin and lack of viable tissue. Distant recurrence was defined as the presentation of new tumors in the untreated portions of the liver or in extrahepatic regions. Follow-up was terminated either at the time of the patient’s death or with the last telephone call through June 1, 2018.

The therapeutic strategy for recurrent tumors was decided using both liver function and tumor backgrounds. RFA should be chosen as the first-line treatment for liver tumors when possible. Combination TACE and RFA therapy or TACE alone is a good choice for treating intrahepatic tumors based on tumor diameter and liver function. If a patient’s Child–Pugh score is over 9 and liver transplantation is impossible, we would recommend palliative care.

### Complications

Complications were estimated based on previously described guidelines for imaging-guided ablation [20]. A major complication was defined as an event that caused substantial morbidity and/or disability, that necessitated an increased level of care, or that required hospital admission or a substantially lengthened hospital stay. All other complications were defined as minor.

### Statistical analysis

Overall survival rates were estimated according to the Kaplan–Meier method. All statistical analyses were performed by SPSS software, version 22.0 (SPSS, Chicago, Ill).

## Results

The included 17 patients had a total of 23 hepatic tumors and 17 tumors adjacent to the second hepatic hilus. The tumor characteristics and patient data are described in Table 1.

**Table 1:** Clinical characteristics and outcomes of study patients

Case	Sex/age	TS (mm)	TN	PVTT	RF session	TACE session	ITR	Survival time (months)
1	M/61	33	2	N	2	2	Complete	18
2	M/47	15	3	N	1	1	Complete	52
3	M/78	23	2	N	1	1	Complete	44
4	M/45	20	1	N	1	1	Complete	29
5	M/54	31	1	N	2	3	Complete	25
6	M/54	22	1	N	2	3	Complete	29
7	M/60	47	1	N	1	1	Complete	27
8	M/33	44	1	N	1	1	Complete	26
9	M/57	48	2	N	2	2	Complete	26
10	F/59	30	1	N	1	1	Incomplete	6
11	M/65	50	1	N	1	2	Complete	26
12	M/61	21	1	N	2	5	Complete	18
13	M/37	28	2	N	2	2	Complete	24
14	M/67	50	1	N	3	3	Incomplete	24
15	M/71	44	1	N	1	1	Complete	19
16	M/63	37	1	N	1	1	Complete	18
17	M/55	38	1	N	1	1	Complete	18

*TS* tumor size, *TN* tumor number, *ITR* initial therapeutic response

Successful placement of RF electrodes was at the site of each tumor, and subsequent RF ablation was completed with a planned protocol in all 17 patients (technical success rate was 100%). Tumor enhancement disappeared with an ablative margin or a sufficient margin of over 0.5 cm after the initial RFA session in 15 of the 17 patients (primary technique effectiveness rates were 88.2%).

### Local and distant tumor progression

No extrahepatic spread was documented during the follow-up period (range 6–52 months). Local tumor progression developed in 1 patient (5.9%), and both local tumor progression and new tumor development were documented in 1 patient (5.9%). The development of new tumors occurred in untreated portions of the liver in 8 patients (47.1%).

Of the 10 patients who experienced local tumor progression or new intrahepatic tumor development, 8 patients underwent a repeat combination therapy of TACE and RFA. 1 patient underwent TACE alone, and the other patient refused any further treatment. After the second treatment procedure, 6 of the 8 retreated patients had further tumor recurrence. Of these 6 patients, 1 patient underwent a third treatment of combined TACE and RFA, 4 patients underwent a third treatment of TACE, and 1 patient subsequently underwent a fourth treatment of TACE.

### Overall and recurrence-free survival

During the follow-up period, 6 of 17 patients (35.3%) died. These deaths were due to tumor progression in 3 patients (17.6%), liver failure in 2 patients (11.8%), and massive gastrointestinal tract hemorrhage in 1 patient (5.9%). 1 patient, who refused any treatment after initial tumor recurrence, died within 6 months due to tumor progression. This patient developed local tumor recurrence 1 month after RFA, and she refused any further treatment, including TACE and radiotherapy. The survival time of 1 patient was 33 months, with death resulting from liver failure.

**Fig. 3:**
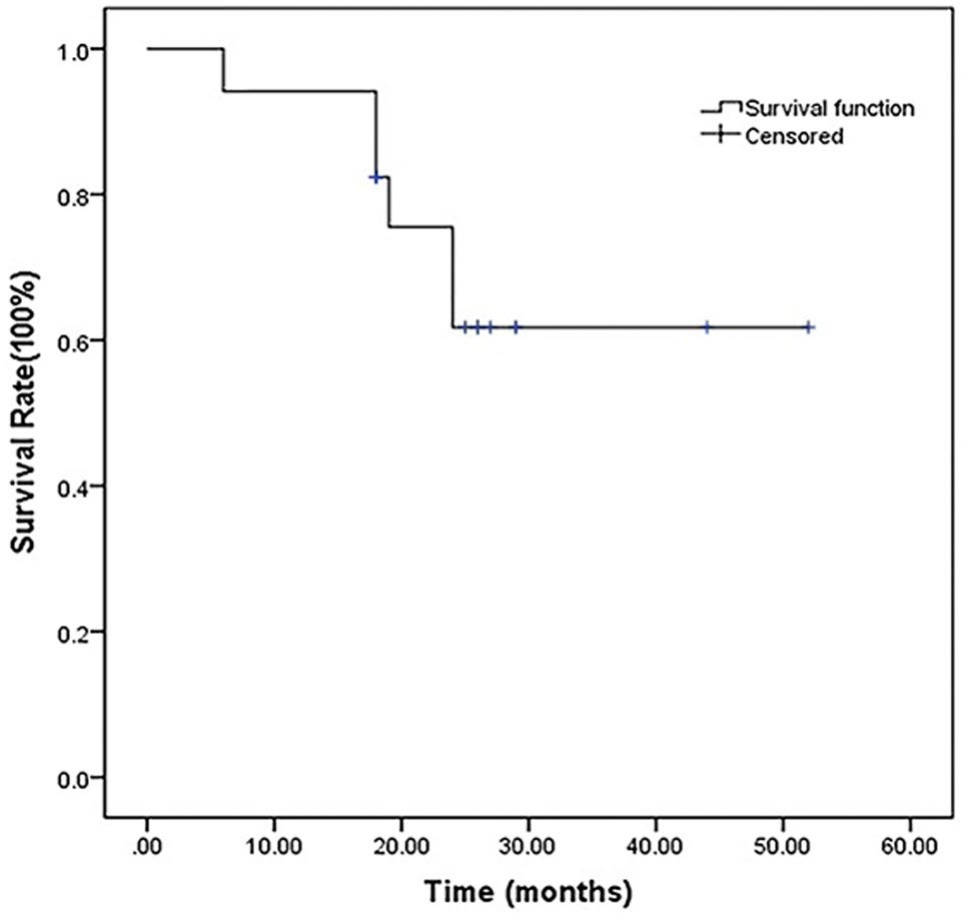
The Kaplan–Meier curves of OS in patients with a tumor adjacent to the second hepatic hilus who underwent combination TACE and RFA therapy. Whole study population *n* = 17, median OS = 25 months, 95% CI = 18–27

The overall survival rates for the 17 study patients were 94.1% (16/17) at 12 months, 82.4% (14/17) at 18 months, and 61.8% (11/17) at 24 months. The median survival time of all 17 patients was 25 months (95% CI 18–27) (Fig. 3).

### Complications

There were no TACE and RFA procedure-related deaths, nor did any major complications such as biliary or hepatic vein complications develop during the treatment procedure.

Minor complications occurred in 5 patients. A self-resolving pneumothorax appeared in 1 patient, a self-resolving subcapsular liver hematoma developed in 1 patient, severe pain occurred in 2 patients, and 1 patient underwent hyperpyrexia 2 days after RFA. Patients with a self-resolving pneumothorax or subcapsular liver hematoma did not require any special treatment or longer-than-normal observation periods. The patient with severe pain was treated by analgesic medicine orally, and the pain disappeared 3 days later. The cause of the single occurrence of hyperpyrexia was confirmed to be bacteremia by hemoculture, and was successfully treated with intravenous antibiotics. All of the patients underwent hepatoprotection treatment after RFA and displayed full recovery of liver function prior to being discharged from the hospital.

## Discussion

Thanks to recent technical developments such as the development of the cluster electrode and the availability of a generator that can deliver increased power to body tissues, the results of RFA for HCC tumors larger than 3–5 cm in diameter may be more successful [22]. Besides, TACE embolization before RAF is more effective in all liver tumors than RAF or TACE treatment alone, especially for tumors larger than 3 cm in diameter, and the intrahepatic lesions after TACE treatment are more likely to be shown during ablation [2, 10]. For some tumors, which are adjacent to critical structures such as near the hilar bile duct and blood vessels adjacent to the second hepatic hilus, the application of a combined therapy of TACE and RFA was considered very difficult. The reason for this difficulty is due to the incomplete ablation resulting from the “heat sink effect,” potentially resulting in serious damage of bile duct or vein from high temperatures. Few papers have reported on the therapeutic outcomes of HCC tumors which are adjacent to the second hepatic hilus. A possible reason for this may be that when a tumor is intertwined with major vessels, it makes it difficult to perform RFA. A standard therapeutic plan for patients with HCC tumors adjacent to the second hepatic hilus remains largely controversial and not well-established.

In our study, complete response was achieved in 15 of 17 patients (88.2%). The result shows that the combination treatment strategy is feasible and effective for patients with HCC adjacent to the second hepatic hilus. In reviewing our results, the reasons for the more successful treatment might include the following: firstly, the method of using CT guidance under general anesthesia appears to help more clearly display the borders of the tumor, and deploying RF electrodes to preplanned sites of tumors adjacent to the second hepatic hilus can allow more precise localization of the treatment site [23, 24]. The TACE procedure can occlude tumor blood flow with a lipiodol injection in liver tumors, which can reduce the heat diffusion through the bloodstream and increase the necrotic volume of the tumor [23]. On the other hand, TACE can also treat the microscopic lesions [25], however, lipiodol deposition may also be lost in the time between procedures, therefore, TACE should be followed by RFA as quickly as possible, to maintain a short interval between the two procedures [26]. In our research we agreed with this viewpoint, and all of our patients underwent the RFA procedure within 7 days of having TACE. Secondly, the CT guidance can provide higher resolution without the interference of gas-release artifacts during the procedure [15], in addition to providing a position of the electrode. The resulting real-time imaging can guarantee that all tines were at the optimal locations, which decreases the likelihood of hurting the nearby blood vessels or bile duct during the procedure. Our study has shown that having the tines of the electrode close to but not in the vein is safe and can accomplish a complete ablation of the HCC tumor.

Peng et al*.* [27] have reported a study including 17 patients, in which RFA was used for the treatment of HCC in the caudate lobe. The 12- and 24-month overall survival rates were 88% and 80%, respectively. The author indicated that a tumor located in the caudate lobe is difficult to treat because it is located deep between the hepatic hilum and the inferior vena cava [27]. A HCC adjacent to the second hepatic hilus also poses a similar problem of higher surgical risk, and also an increased risk of damaging the vessel or bile duct during RFA. Compared with the caudate lobe tumor ablation study, the overall survival rate in our study was higher with fewer complications, one reason is attributed to the combined treatment of TACE and RFA along with the usage of the cluster electrode, another one is that the number of cases is too small.

Yang et al. [16] recently reported the use of ultrasound-guided RFA to treat HCC in difficult locations. The complication rate seen in tumors in more difficult locations was higher than that in those in more easily accessed locations (4.9% vs 0.8%, *P* = 0.041). Severe complications occurred in 29 patients, and included hemoperitoneum, biliary injury, hemothorax, pyothorax, liver abscess, intestinal perforation, cholecystitis, needle-track seeding, and intestinal perforation-related death. By contrast, our study showed higher safety, no serious complications, and only minor complications developed in 5 patients. We attribute the low rate of complications to our method of using CT guidance, general anesthesia, and a multiple-tined electrode.

Our study does have some limitations. First, as it was a retrospective study, there might be a potential risk for selection bias. Second, we only used the cluster RF electrode and not the singular RF electrode in our current study, so we do not know if there is a difference in the effectiveness and safety between the two types of the electrode for the treatment of a second hepatic hilus tumor. Lastly, this study may have inherent bias associated with the small sample size and being located in a single study center. Thus, large prospective and multi-center studies should be performed in the future to establish the efficacy of RFA combined with TACE in the treatment of HCC located adjacent to the second hepatic hilus.

## Conclusion

In conclusion, HCC which is adjacent to the second hepatic hilus, or adjacent to the main bile duct and blood vessels should not be considered an absolute contraindication for RFA treatment. The combination therapy of TACE and RFA appears to be an effective therapeutic strategy, especially via the technique of placing the electrode tines close to but not in the vein. With careful pretreatment planning and accurate measurements, with judicious patient selection, and by performing the treatment course with CT guidance, complete ablation of the tumor in an atypical location can be successfully achieved.

## Data Availability

The datasets used and/or analyzed during the current study are available from the corresponding author upon reasonable request.
